# Role of Prefrontal Persistent Activity in Working Memory

**DOI:** 10.3389/fnsys.2015.00181

**Published:** 2016-01-05

**Authors:** Mitchell R. Riley, Christos Constantinidis

**Affiliations:** Department of Neurobiology and Anatomy, Wake Forest School of MedicineWinston-Salem, NC, USA

**Keywords:** prefrontal cortex, monkey, neurophysiology, fMRI, neuron

## Abstract

The prefrontal cortex is activated during working memory, as evidenced by fMRI results in human studies and neurophysiological recordings in animal models. Persistent activity during the delay period of working memory tasks, after the offset of stimuli that subjects are required to remember, has traditionally been thought of as the neural correlate of working memory. In the last few years several findings have cast doubt on the role of this activity. By some accounts, activity in other brain areas, such as the primary visual and posterior parietal cortex, is a better predictor of information maintained in visual working memory and working memory performance; dynamic patterns of activity may convey information without requiring persistent activity at all; and prefrontal neurons may be ill-suited to represent non-spatial information about the features and identity of remembered stimuli. Alternative interpretations about the role of the prefrontal cortex have thus been suggested, such as that it provides a top-down control of information represented in other brain areas, rather than maintaining a working memory trace itself. Here we review evidence for and against the role of prefrontal persistent activity, with a focus on visual neurophysiology. We show that persistent activity predicts behavioral parameters precisely in working memory tasks. We illustrate that prefrontal cortex represents features of stimuli other than their spatial location, and that this information is largely absent from early cortical areas during working memory. We examine memory models not dependent on persistent activity, and conclude that each of those models could mediate only a limited range of memory-dependent behaviors. We review activity decoded from brain areas other than the prefrontal cortex during working memory and demonstrate that these areas alone cannot mediate working memory maintenance, particularly in the presence of distractors. We finally discuss the discrepancy between BOLD activation and spiking activity findings, and point out that fMRI methods do not currently have the spatial resolution necessary to decode information within the prefrontal cortex, which is likely organized at the micrometer scale. Therefore, we make the case that prefrontal persistent activity is both necessary and sufficient for the maintenance of information in working memory.

## Introduction

Working memory is the ability to maintain and manipulate information in mind, over a time span of seconds (Baddeley, 2012). The memory system storing information for a few seconds was termed “short-term memory” in the classical, three-store model of memory (Atkinson and Shiffrin, 1968). The modern definition of working memory emphasizes its dynamic nature of representing and manipulating information originating from the environment or retrieved from long-term memory, rather than being a passive conduit of information into the long-term memory store (Baddeley, 2003; Smith and Kosslyn, 2007). In recent years, some authors have reserved the term “working memory” to refer specifically to complex information that needs to be manipulated; the term “visual short term memory” has been used to denote memory of simple stimuli (e.g., colored squares) that needs to be maintained without any further transformation (Todd and Marois, 2004). Although important in its own right, working memory is a core component of a number of other cognitive functions, including language, problem solving, reasoning, and abstract thought (Baddeley, 1992). Its central role in cognitive function explains the intense research interest that spans several decades.

**Figure 1:**
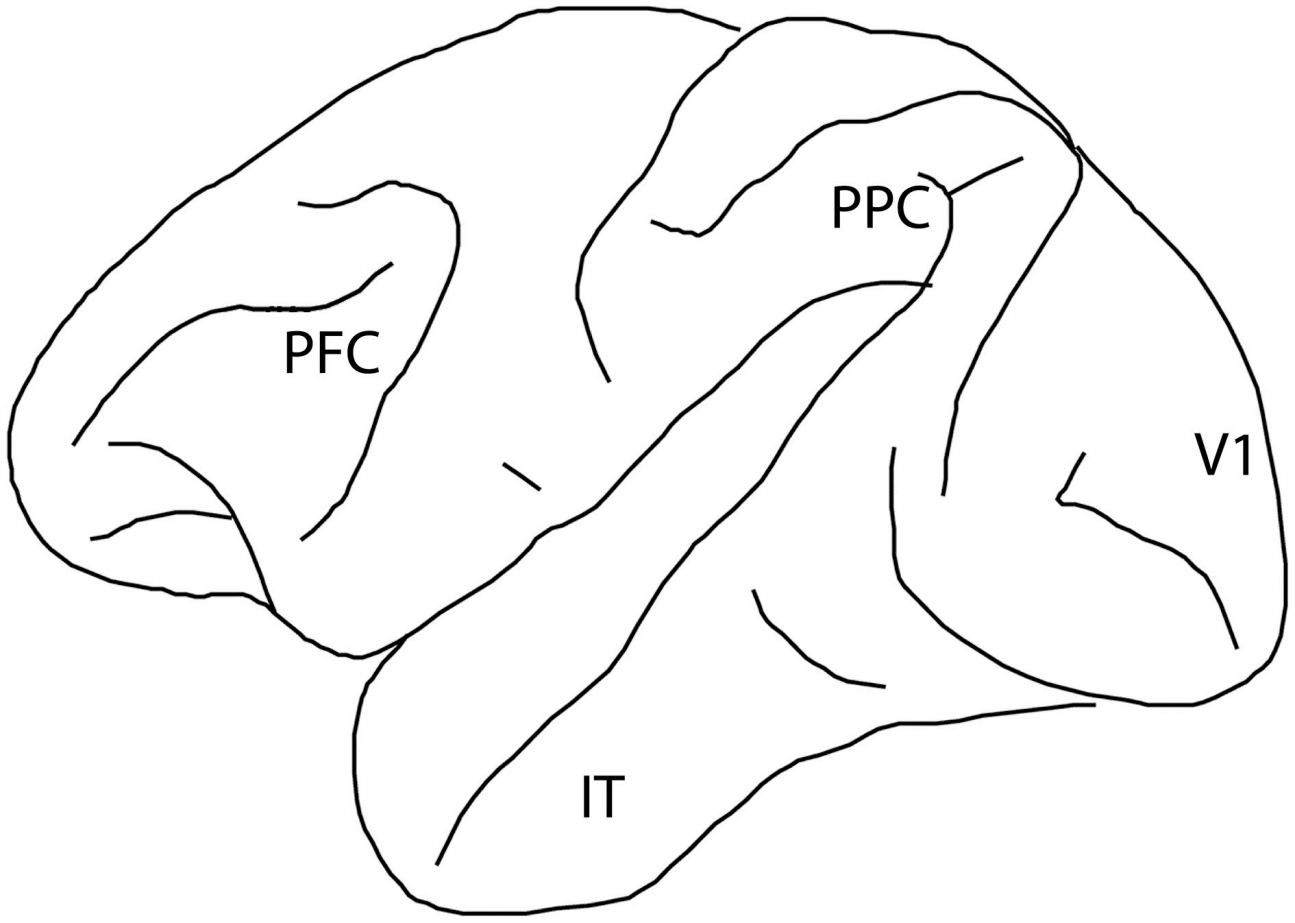
**Diagram of the monkey brain, with four cortical regions implicated in visual working memory labeled: prefrontal cortex (PFC), posterior parietal cortex (PPC), primary visual cortex (V1), and inferior temporal cortex (IT)**.

Studies of lesions in humans and non-human primates first implicated the cortical surface of the frontal lobe as the site of working memory function (Jacobsen, 1936; Milner, 1963). Lesions of the prefrontal cortex (PFC—Figure 1) rendered subjects unable to perform even simple tasks requiring working memory. A wide range of impairments in tasks requiring manipulation of information in memory has been confirmed in recent lesion studies (Rossi et al., 2007; Buckley et al., 2009). Subsequently, neurophysiological experiments identified neurons that not only respond to sensory stimuli, but remain active during a period after a stimulus was no longer present; this “persistent activity” therefore provided a neural correlate of working memory (Fuster and Alexander, 1971; Funahashi et al., 1989). Visuo-spatial working memory has been a particularly fruitful model since spatial location can be varied parametrically and the activity of neurons representing each location can be studied systematically. Persistent activity in the prefrontal cortex has been shown to explain many aspects of behavioral performance in visuo-spatial working memory tasks (Qi et al., 2015b).

The role of prefrontal cortex in working memory has been re-evaluated over the past few years (Sreenivasan et al., 2014a; D'Esposito and Postle, 2015) as several sources of experimental evidence have challenged the traditional views on prefrontal persistent activity. First, neurophysiological studies have demonstrated that persistent discharges are not limited to the prefrontal cortex, but are widespread in a network of cortical and subcortical areas, thus raising questions on the role of persistent firing in the prefrontal cortex (Constantinidis and Procyk, 2004; Pasternak and Greenlee, 2005). Secondly, phenomena such as repetition suppression illustrate that the activity of neurons may be modulated by prior stimuli in the absence of persistent activity (Grill-Spector et al., 2006). Third, human fMRI studies have been successful in decoding information held in memory from visual cortex (Harrison and Tong, 2009) and have identified correlates of working memory capacity in the posterior parietal cortex (Todd and Marois, 2004, 2005; Xu and Chun, 2006). Therefore, alternative models based on interpretation of BOLD signals (which do not directly measure spiking activity) ascribe control processes to PFC while reserving the representation of working memory for the sensory cortices (Curtis and D'esposito, 2003; D'Esposito and Postle, 2015).

In this review, we examine the role of prefrontal cortex in working memory. We take a position largely in favor of the classical model of working memory being represented in the persistent activity of prefrontal neurons based on evidence from neurophysiological experiments in non-human primates and critical evaluation of human imaging studies. We begin by examining the anatomical basis of working memory and the specializations of the prefrontal cortical circuit. We then review the range of phenomena accounted for by persistent activity in visuo-spatial working memory, illustrating the enduring appeal of the model. Activation during spatial working memory may be viewed as equivocal about the role of the prefrontal cortex because persistent activity might be explained by top-down control processes as well as by working memory itself. We therefore discuss the evidence of prefrontal persistent activity for other content types of working memory. We then review memory models not dependent on persistent activity and posit that these could only mediate a limited range of working memory tasks. We finally review activity decoded from brain areas other than the prefrontal cortex during working memory, concluding that the ultimate source of this activation is the prefrontal cortex, and these areas alone are not sufficient for mediating working memory maintenance.

## Anatomical organization of working memory circuits

**Figure 2:**
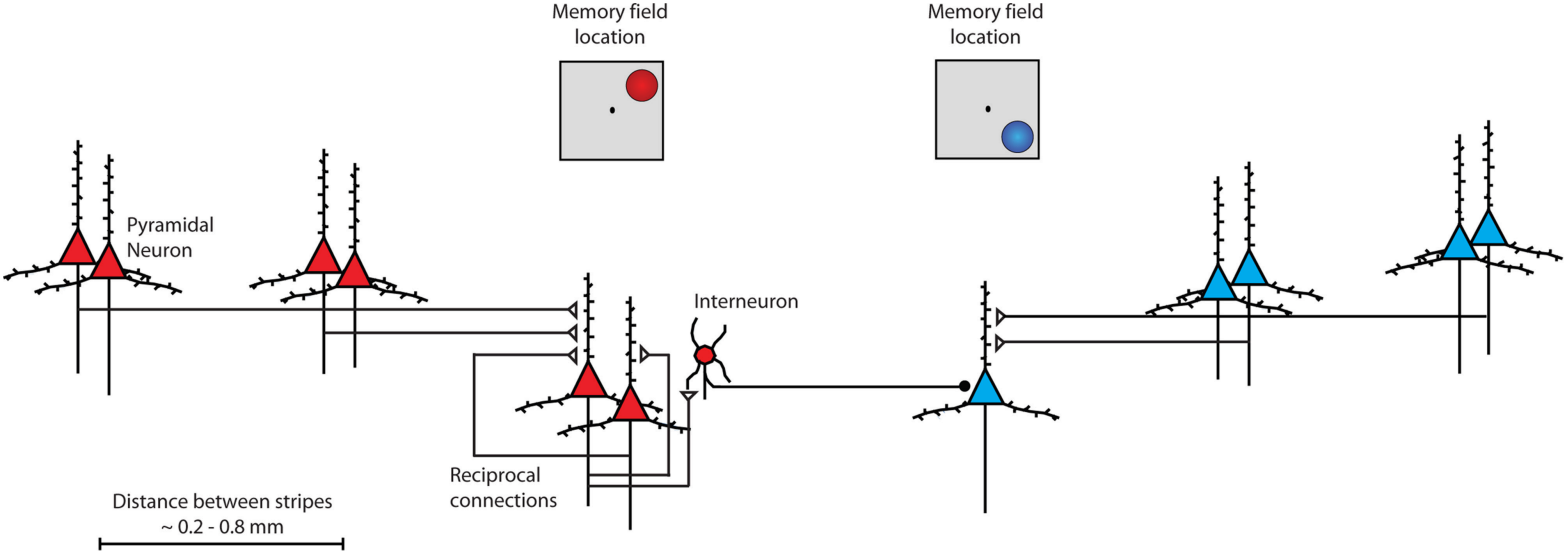
**Schematic diagram of intrinsic connections between neurons within the prefrontal cortex**. Neurons with similar tuning (memory field representing upper right location) are drawn in red color. Pyramidal neurons excite each other through reciprocal connections. Stripes of neurons with similar spatial tuning are repeated across the surface of the cortex. Interneurons inhibit other pyramidal neurons with different spatial tuning (memory field representing lower right location) drawn in blue color.

To understand why prefrontal cortex may represent robustly remembered information, it is instructive to review the anatomical basis of persistent activity. The primary source of sustained excitation is thought to be reverberating activity through layer II/III horizontal excitatory connections between prefrontal neurons with similar stimulus tuning (Constantinidis and Wang, 2004). PFC neurons receive horizontal connections from clusters of cells (Figure 2), arranged in stripe-like fashion, 0.2–0.8 mm wide (Goldman-Rakic, 1984; Levitt et al., 1993; Lund and Lewis, 1993; Kritzer and Goldman-Rakic, 1995; Pucak et al., 1996). Persistent firing between layer II/III neurons also depends on glutamate stimulating NMDA receptors (Wang et al., 2013). The relatively slow time constant of NMDA receptors allows the post-synaptic neuron to remain at a relatively depolarized state for a longer interval, compared to neurons containing AMPA receptors alone; without NMDA receptors, an unrealistically high level of firing rate would be required to sustain persistent activity (Wang, 2001). Additionally, sharper tuning for spatial location arises from GABAergic interneurons, which are essential in tuning the activity to represent specific spatial information (Rao et al., 1999, 2000; Constantinidis and Goldman-Rakic, 2002).

Several anatomical specializations endow the prefrontal cortex with unique properties in maintaining persistent activity. Prefrontal pyramidal neurons exhibit the most extensive dendritic trees and highest number of spines of any cortical neurons, some 23 times higher than the number of spines of layer III pyramidal cells in V1 (Elston, 2000, 2003). As a consequence, the spatial spread of functional interactions between neurons within the prefrontal cortex is more extensive than of neurons within the posterior parietal cortex (Katsuki et al., 2014). Additionally, dopaminergic innervation terminates predominantly in the frontal lobe and can improve the signal-to-noise ratio of persistent activity, mainly via enhancement of the NMDA conductance (Yang and Seamans, 1996; Durstewitz et al., 2000; Seamans et al., 2001; Chen et al., 2004). Specialized GABAergic types have also been implicated in stabilizing persistent activity in the face of distraction, and physiological signatures of these neurons have been specifically identified in the prefrontal cortex (Wang et al., 2004; Zhou et al., 2012). All of these specializations suggest that the prefrontal cortex is better suited to generate and sustain persistent activity than its afferent areas (Qi et al., 2015b).

## Persistent activity in visuo-spatial working memory

**Figure 3:**
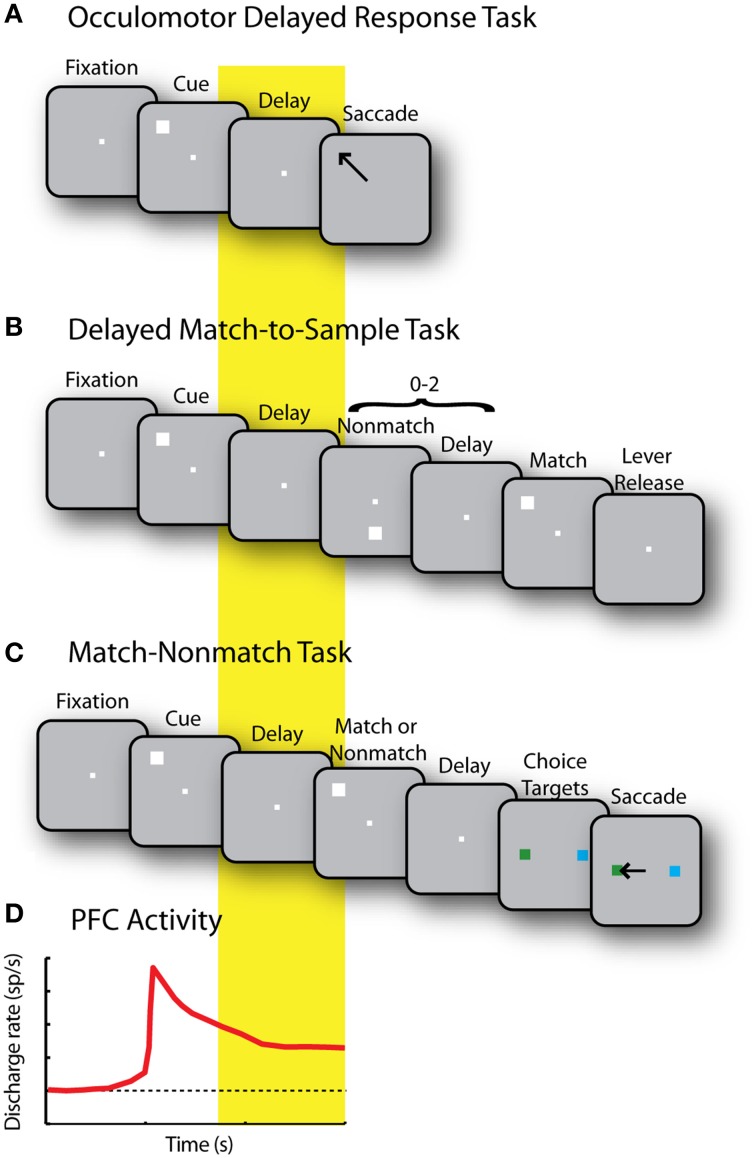
**(A)** Sequence of events in the Oculomotor Delayed Response (ODR) task. Successive frames represent the fixation period, stimulus presentation, delay period, and saccade toward the remembered stimulus location. **(B)** Delayed Match to Sample task. Monkeys first foveate the fixation point and pull a lever. They are then presented with a cue stimulus. This is followed by a random (0–2) number of non-match stimuli, separated by delay periods. When a match stimulus appears at the same location as the cue, the monkeys are required to release the lever. **(C)** Match/Non-match task. While monkeys fixate, two stimuli are presented in sequence, separated by delay periods. After another delay period, two choice targets are shown and the monkey has to saccade to the green target if the second stimulus matched the cue, and the blue stimulus, otherwise. **(D)** Schematic diagram of prefrontal activity elicited by the stimulus that is sustained during the delay period in each of the previous tasks.

The most extensively used paradigm to study visuo-spatial working memory involves the oculomotor delayed response (ODR) task (Figure 3A), which presents subjects with a brief stimulus and, after a delay period, requires an eye movement to its remembered location (Funahashi et al., 1989; Rao et al., 1999; Constantinidis et al., 2001a). Another common task, the delayed alternation task, similarly requires a (hand or eye) movement to one of two locations, alternating in successive trials, therefore requiring memory for the location of the preceding choice (Kubota and Niki, 1971; Niki, 1974). Persistent activity selective for the spatial location of the remembered stimulus is apparent in a population of prefrontal neurons, comprising approximately a third of the total prefrontal neurons (Qi and Constantinidis, 2013). The location of the preceding stimulus in such tasks is sometimes confounded with the preparation for the motor response; however, more complex tasks reveal that the majority of prefrontal neurons represent the former rather than the latter. For example, when a task requires monkeys to make an eye movement toward a location other than the location of the visual stimulus, the majority of prefrontal neurons represent the location of the preceding stimulus rather than the location of the impeding saccade. This is the case in the delayed anti-saccade task (Funahashi et al., 1993b) and the rotational ODR task (Takeda and Funahashi, 2002).

A recent study revives the idea that persistent activity generated during ODR tasks represents motor preparation rather than memory for the stimulus (Markowitz et al., 2015). The study used two versions of the ODR task, one in which the stimulus appeared transiently (as in Figure 3A) and one in which it remained visible for the entire interval until the motor response. The conclusion that persistent activity represents motor preparation was predicated entirely on the assumption that memory storage is only mediated by neurons that exhibit persistent activity after the stimulus has been turned off, but do not continue to respond to the stimulus when it remains visible. Neurons exhibiting continuous activation by visual stimuli were considered “preparation” neurons, by default. This premise is tenuous. Neither direct evidence nor network models are available that would suggest that memory storage neurons are not activated continuously by a prolonged stimulus. In turn, this assumption leads to the conclusion that the activity of “storage units,” thus defined, has no influence on recall performance or other aspects of behavior in a memory task (Markowitz et al., 2015). This is a questionable conclusion, in our view.

Persistent activity tuned for the location of a stimulus appears in the prefrontal cortex even in tasks where the stimulus does not immediately allow planning of a movement. In the spatial delayed-match-to-sample task, subjects are required to release a lever or press a button when a stimulus appears at a previously cued location (Figure 3B); in the match/non-match task, the monkeys have to saccade to a green or blue response target depending on whether two stimuli presented in sequence appeared at the same location or not (Figure 3C). In such tasks, prefrontal neurons generate persistent activity following the presentation of the original stimulus that is tuned for its spatial location (Figure 3D), and not the preparation of a motor response, the direction of which is not known until later in the trial (Qi et al., 2010, 2011; Goodwin et al., 2012).

Persistent activity is not merely an epiphenomenon of spatial working memory, either. The most straightforward evidence in favor of this idea comes from analysis of error trials in the ODR task, which are characterized by lower levels of delay period activity (Funahashi et al., 1989; Zhou et al., 2013). In other words, trials in which persistent activity is diminished are more likely to result in errors. A near linear relationship between behavioral performance and persistent activity can be also revealed in tasks that modulate parametrically the discriminability of two remembered targets (Constantinidis et al., 2001b).

**Figure 4:**
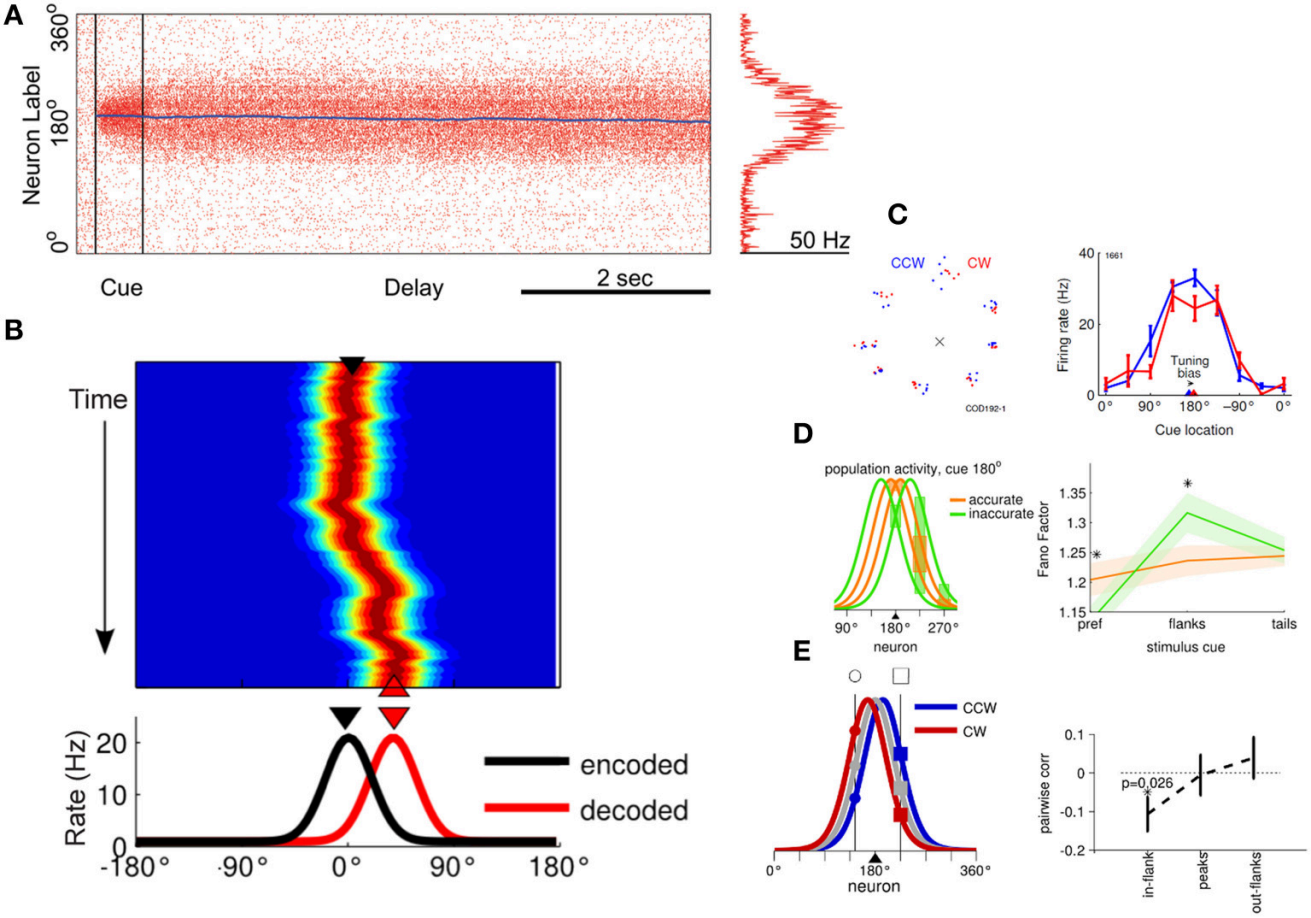
**(A)** Simulated, network activity in the ODR task, following presentation of a cue at the 180° location. Abscissa represents time during the trial; ordinate represents different neurons arranged based on their tuning. **(B)** Network activity illustrating drifts in the peak of activation during the delay period. Axes have been rotated relative to **(A)**. Color represents firing rate. The black triangle represents the cue position at the beginning of the delay period (encoded population activity on the bottom graph). The red triangle represents the location decoded by the population activity at the end of the delay period. **(C)** Left, saccade endpoints in one behavioral session divided into trials that landed clockwise (red) or counterclockwise (blue) relative to the cue stimulus position. Right, delay-period responses of one neuron recorded during the same session. The triangles indicate the circular mean of the tuning curve obtained from trials that generated clockwise, or counterclockwise saccadic deviations. **(D)** Left, schematic representation of four different delay period population activity profiles to the same 180° cue. Red lines represent trials with saccadic endpoints closer to the target (accurate trials) and green lines represent trials farther from the target (inaccurate trials). Right, difference between discharge variability in inaccurate and accurate trials depending on the location of the cue. Variability is maximal for cue appearing at the flanks of the neuron's tuning curve, where small deviations cause large differences in firing rate. **(E)** Left, schematic representation of delay period activity of two neurons recorded simultaneously, whose tuning peaks lie at opposite sides of the activity bump. Right, trial-to-trial correlations are negative between these neurons as a bump in activity leads to an increase in firing rate of one neuron with a decrease in the other neuron. Panel **(A)** adapted with permission from Renart et al. (2003); panels **(B–E)** from Wimmer et al. (2014).

Computational models provide a detailed picture of the relationship between behavioral outcomes related to working memory performance and persistent activity (Figure 4). Persistent activity can be sustained in such models by virtue of re-entrant connections between neurons with similar tuning for stimulus properties, so that activation after afferent input is maintained in the system (Figure 4A). Drifts in neuronal activity across the network of prefrontal neurons (Figure 4B) have been shown to predict precisely the relationship between several aspects of firing rate and the endpoint of the saccade (the spatial location being recalled by the monkey) in the ODR task (Wimmer et al., 2014). For example, persistent activity recorded from trials in which monkeys make eye movements deviating clockwise vs. counterclockwise relative to the true location of the stimulus yields slightly different tuning curves, as would be expected if the location recalled was determined by the peak of activity at the end of the delay period (Figure 4C). Similarly, the variability of a neuron's delay period activity (estimated by the Fano factor of spike counts, i.e., the variance divided by the mean) is maximal for inaccurate saccades to locations at the flanks of the neuron's tuning curve but lower for locations in the peak or tail (Figure 4D). This counterintuitive finding is also explained if one appreciates that small deviations in saccadic endpoint correspond to the bump of activity shifting in one direction or another, and that activity of a single neuron changes most rapidly if the bump traverses the flank of its tuning curve rather than its peak or tail. Finally, spike-count correlations of two simultaneously recorded neurons are lowest and negative for inaccurate saccades when the cue appears between the peaks of their tuning curves (Figure 4E). This result is also consistent with the idea that working memory inaccuracies are caused by drifts of persistent activity in the delay period, and when the bump attractor randomly varies around a location between the peaks of two neurons, it inevitably causes an increase in firing rate for one neuron, but a decrease for the other. Importantly, these findings do not hold for neurons that do not exhibit persistent discharges, even though the latter are more numerous in the prefrontal cortex (Wimmer et al., 2014).

Persistent activity in the prefrontal cortex has also been shown to be subject to developmental changes, with lower levels of persistent activity present in older monkeys (Wang et al., 2011). This decline has been linked to alpha-adrenergic receptors. Drugs targeting these can ameliorate the effects of age-related cognitive deficits (Arnsten and Goldman-Rakic, 1985; Arnsten et al., 1988), as well as increase persistent discharges to levels seen in younger adults (Wang et al., 2011). An important concept to consider is that persistent activity is not the same as a generalized increase in neuronal excitability. For example, low doses of a nicotinic alpha-7 agonist enhance spatially tuned persistent activity but high doses produce non-specific excitation that erodes the representation of the remembered spatial location (Arnsten and Wang, 2016).

## Persistent activity in non-spatial working memory

Prefrontal neurons generate discharges that represent other types of information, in addition to spatial location. Ventrolateral prefrontal cortex receives input from regions of the ventral visual pathway, most importantly the inferior temporal cortex and superior temporal gyrus (Petrides and Pandya, 1988; Webster et al., 1994). Generally, smaller populations of prefrontal neurons are tuned for object attributes such as geometric shape, color, or complex features (e.g., specific faces), than spatial location; a regional specialization is also present, with spatial information more prevalent in the dorsolateral prefrontal cortex than the ventrolateral prefrontal cortex (Meyer et al., 2011). Nonetheless, robust, stimulus-selective persistent activity has been described in working memory tasks requiring subjects to remember the identity and features of stimuli. Examples include stimuli defined by simple, geometric shapes differing in color or luminance (Quintana et al., 1988; Hoshi et al., 1998; Constantinidis et al., 2001b; Sakagami et al., 2001; Averbeck et al., 2003; Inoue and Mikami, 2006; Genovesio et al., 2009), complex images, such as real objects and faces, or abstract pictures (Wilson et al., 1993; Miller et al., 1996; O Scalaidhe et al., 1997, 1999; Rao et al., 1997; Rainer et al., 1998; Rainer and Miller, 2000; Freedman et al., 2001; Roy et al., 2014) and the direction of motion of a random-dot stimulus that is always presented at the same location (Zaksas and Pasternak, 2006; Mendoza-Halliday et al., 2014).

In recent years, it has been recognized that persistent activity in the prefrontal cortex also represents information beyond the characteristics of stimuli. Activity may represent the abstract rules of the cognitive task subjects are required to perform (White and Wise, 1999; Wallis et al., 2001), categories (Freedman et al., 2001; Shima et al., 2007), and numerical quantities (Nieder et al., 2002). It may be also related to perceptual decisions (Kim and Shadlen, 1999; Barraclough et al., 2004), reward expectation (Leon and Shadlen, 1999), and sequences of events or actions (Averbeck et al., 2002; Inoue and Mikami, 2006; Sigala et al., 2008; Berdyyeva and Olson, 2010). Persistent activity of single neurons may represent more information than stimulus features and task variables simultaneously (Rigotti et al., 2013). For instance, persistent firing may represent different aspects of the task demands as they change over time, thus providing dynamic representations (Mante et al., 2013).

The realization that prefrontal activity is modulated by task factors to such extent has led to a re-evaluation of the nature of information represented in persistent activity (D'Esposito and Postle, 2015). Taken to the extreme, this idea would suggest that all stimulus-selective information that appears to be represented in the prefrontal cortex is in fact related to task rules or categorical judgments between alternatives rather than representing the memoranda themselves. In an attempt to pinpoint the nature of information represented in the prefrontal cortex, some experiments have relied on working memory for stimuli defined solely by elemental properties, such as direction of motion or color, and found the ability of prefrontal cortex to represent such features wanting. In an experiment requiring subjects to remember the overall direction of motion of an initial random-dot display and decide if the direction of a following display was the same or different, prefrontal neurons exhibited only transient representation of direction information in the delay period (Zaksas and Pasternak, 2006). Another experiment that required memory for the color of a stimulus revealed that very few prefrontal neurons exhibited pure color information, as opposed to information about its location (Lara and Wallis, 2014).

Ruling out prefrontal cortex as the cortical area mediating the representation of object information in working memory based on such negative findings appears premature. More recent experiments have succeeded in revealing robust persistent activity representing direction of motion throughout the delay period of a working memory task in the prefrontal cortex (and area MST) but not in area MT of the visual cortex, although MT was robustly activated during the presentation of these stimuli (Mendoza-Halliday et al., 2014). In the case of color, too, activation of only a small proportion of prefrontal neurons, in the order of 5–15% (Lara and Wallis, 2014) may be sufficient for the representation of stimulus information. It is also possible that color-selective neurons are concentrated in specific prefrontal “patches” (Lafer-Sousa and Conway, 2013) and persistent activity representing color information may be concentrated in such modules rather than be diffused across the entire prefrontal surface.

Persistent neuronal firing in prefrontal cortex has been observed even in the absence of performance of a task, or even learning of a task, while subjects view stimuli, passively. Prefrontal neurons have thus been shown to generate persistent discharges tuned for stimulus location and shape in monkeys never trained to perform a working memory (or other cognitive) task (Meyer et al., 2011; Meyers et al., 2012). The fact that prefrontal neurons generate persistent activity when not required to perform a working memory task is not incompatible with our intuition of working memory, either. We are able to recall stimuli we encounter even when we are not prompted to maintain them in memory ahead of time (Qi et al., 2015b). Consistent with this finding, recordings during passive fixation reveal persistent discharges selective for faces in the ventrolateral prefrontal cortex (O Scalaidhe et al., 1999). Prefrontal neurons also represent stimulus features even when they are irrelevant for the task at hand (Constantinidis et al., 2001b; Lauwereyns et al., 2001; Donahue and Lee, 2015). This evidence argues that persistent activity in the prefrontal cortex is sufficient to represent object-related information in working memory. In Section Alternative Working Memory Models, we will review the evidence that prefrontal cortex is also necessary for this role.

## Alternative working memory models

In recent years, the role of persistent activity has come into question by alternative models proposed to mediate working memory. By some accounts, information can be maintained in memory over a period of seconds through mechanisms other than persistent discharges. We will examine three categories of models here: non-spiking models dependent on synaptic mechanisms, rhythmic-spiking models conveying information based on the frequency and phase of discharges without necessarily an increase in overall activity, and dynamic-spiking models in which information is represented based on the pattern of neurons that are active without an elevation of mean firing rate across the population.

### Non-spiking models

Activity elicited after repeated presentation of the same stimulus is typically reduced, a phenomenon termed repetition suppression (Grill-Spector et al., 2006). As a result, the level of response to a particular stimulus in the context of a working memory task, such as the delayed match to sample task, can be informative about whether it was preceded by the same stimulus or not; match suppression may signal that the sample was the same as the match. This suppressed response to a matching stimulus is observed even though several seconds may intervene between the sample and match, and it does not require persistent activity (Miller et al., 1991, 1996). Match suppression (or enhancement, for some neurons) is observed for stimuli matching in shape, color, and form, in spatial location, or in direction of motion, in various cortical areas, including the prefrontal, posterior parietal, and inferior temporal cortex (Miller et al., 1991, 1996; Steinmetz et al., 1994; Zaksas and Pasternak, 2006; Woloszyn and Sheinberg, 2009). Furthermore, the extent of response difference to matching and non-matching stimuli has predictive power over behavioral performance, as it differs systematically in correct and error trials (Zaksas and Pasternak, 2006; Qi et al., 2012).

Computational models have been proposed that could account for such changes via mechanisms that do not depend on spike generation, but instead involve modification of synaptic strengths (Mongillo et al., 2008; Sugase-Miyamoto et al., 2008). Such mechanisms may be mediated by calcium availability at the presynaptic terminal, whose kinetics have a time constant in the scale of seconds (Mongillo et al., 2008). The duration and stability of working memory in such models may still be modulated by spiking activity.

Repetition suppression is a robust phenomenon observed across multiple cortical areas and the fact that the match/non-match effect differs in correct and error trials offers compelling evidence that memory performance has access to this activity. However, it is a phenomenon limited to recognition memory that may not even mediate representation of the identity of the remembered stimulus, and it cannot account for working memory performance in other tasks. It is hard to imagine an equivalent role of synaptic mechanisms for tasks such as the ODR, delayed alternation, N-back, or free recall tasks. Moreover, other computational models show that even though preference for a non-match over a match stimulus may be present in individual neurons with no persistent activity, the phenomenon may still be mediated by a network that depends on persistent activity (Engel and Wang, 2011). It is still an open question, therefore if synaptic mechanisms have a role in working memory in the absence of persistent activity.

### Oscillatory models

Rhythmic activity has long been implicated in hippocampal-dependent memory, and communication between the hippocampus and prefrontal cortex, in rodents (Buzsaki, 2010). In the human literature, the frequency of oscillations evident through MEG, EEG, and ECoG recordings has also been associated with distinct working memory processes (Roux and Uhlhaas, 2014). Recent neurophysiological studies in non-human primates have begun to address more specifically what role rhythmic firing may play in working memory (Siegel et al., 2009; Buschman et al., 2012; Liebe et al., 2012; Salazar et al., 2012; Brincat and Miller, 2015). The magnitude, frequency, and phase of oscillations within the prefrontal cortex and between the prefrontal cortex and other areas have been shown to be modulated depending on stimulus and task information (Buschman et al., 2012; Liebe et al., 2012). Therefore, information about the stimulus held in memory or task to be performed may be decoded based on these parameters. For example, oscillatory synchronization between LFP signals recorded from different sites within the prefrontal cortex has been shown to be modulated based on which of two task rules a monkey is performing (Buschman et al., 2012). The coherence in rhythmic synchronization between neurons in prefrontal and posterior parietal cortex has also been reported to be content dependent; in other words, prefrontal and parietal neurons synchronize their firing at specific frequencies, for different stimuli held in memory (Salazar et al., 2012). The phase of rhythmic activity could also differentiate information representing two sequentially presented stimuli (Siegel et al., 2009).

Oscillatory activity is not incompatible with persistent activity. For example, both robust persistent activity and gamma-band rhythmicity have been reported during the delay period of the ODR task (Pesaran et al., 2002), as well as the two-item memory task described above (Siegel et al., 2009). It is an open question whether oscillatory activity may dictate behavioral performance in working memory tasks independently of persistent activity.

### Dynamic information models

Information may be represented dynamically in a neuronal population without having to be rhythmic. The precise pattern of activation of different neurons at each time point during a working memory task can be used to decode the identity of the stimulus, even though overall activity during the delay period is not significantly elevated above the baseline (Stokes et al., 2013). This result provides yet another alternative mechanism of working memory representation.

The existence of stimulus information that can be decoded by the dynamic pattern of activation in the prefrontal population (Stokes et al., 2013) presents challenges to the persistent activity model. We should consider however that the stimuli used in the Stokes et al. study are similar to those used in previous studies where persistent activity was observed (Miller et al., 1996; Rao et al., 1997; Rainer et al., 1998). It is possible therefore that a population of neurons did generate persistent activity but might have been too weak to detect when all neurons were averaged together. The demonstration of a condition where persistent activity is truly absent and information is encoded solely by the dynamic pattern of information in neurons whose activity is not modulated during working memory is an open question. Furthermore, dynamic firing models have yet to establish what aspects of information that can be decoded from the dynamic representation of stimulus information can predict behavioral variables, such as recall error rates, accuracy of recall, or reaction time, to the extent that models of persistent activity have been successful in doing (Wimmer et al., 2014).

Dynamic patterns of activation across the population of neurons are not mutually exclusive with persistent activity either. Dynamic activity informative about stimulus identity and task rules has been observed even when persistent activity is present in the population (Crowe et al., 2008; Meyers et al., 2012). Different populations of neurons may also be active at different time points of the ODR task representing stimulus attributes or response preparation (Markowitz et al., 2015). One possible resolution to the two seemingly incompatible mechanisms of information representation is found by analyzing the neuronal population activity during the ODR task. Principal Component Analysis reveals a dynamic, low-dimensional representation, where stimulus location evolves dynamically in time after the cue presentation, but different locations remain constrained in separable subspaces (Roy et al., 2013). Persistent firing specific for the location of a stimulus may thus sweep the population of neurons, in a specific pattern, during the time course of a trial.

## Role of other areas in working memory

Persistent discharges are not an exclusive property of the prefrontal cortex. Neurons in premotor, parietal, cingulate, and temporal association areas generate robust persistent activity, as do subcortical structures including the basal ganglia and the mediodorsal nucleus of the thalamus (Constantinidis and Procyk, 2004; Pasternak and Greenlee, 2005). The proposed alternative mechanisms of memory maintenance reviewed before, and fMRI findings in humans have expanded the list of potential sites of memory into even more cortical areas, as early as the primary visual cortex (Harrison and Tong, 2009). We will next review the evidence of working memory representation in the posterior parietal and inferior temporal cortex (for spatial and object memory, respectively), and in visual cortical areas, including V1.

### Posterior parietal (PPC) and inferior temporal (IT) cortex

The posterior parietal and inferior temporal cortex represent the two main cortical afferents of the prefrontal cortex, as they are strongly interconnected with the dorsolateral and ventrolateral prefrontal cortex, respectively (Constantinidis and Procyk, 2004). Posterior parietal and dorsolateral prefrontal cortex share many functional properties with respect to spatial working memory (Rawley and Constantinidis, 2009) and both regions are activated simultaneously in human imaging studies of working memory (Jonides et al., 1993; Courtney et al., 1997; Owen et al., 1998; Ungerleider et al., 1998; Marshuetz et al., 2000; Bunge et al., 2001; Stern et al., 2001). Neurons in posterior parietal cortex also generate persistent activity (Gnadt and Andersen, 1988), and this has been shown to represent the remembered locations of visual stimuli, independent of a planned motor response (Constantinidis and Steinmetz, 1996). Tested with the ODR task, virtually identical percentages of neurons exhibiting working memory responses were observed in posterior parietal and dorsolateral prefrontal areas (Chafee and Goldman-Rakic, 1998).

Responses of IT neurons related to object memory exhibit many intriguing parallels with spatial working memory in the posterior parietal cortex. IT cortex shares a number of physiological properties with ventrolateral prefrontal cortex and both exhibit memory-related activation. IT neurons discharge in a persistent fashion after the offset of visual stimuli and their activity encodes the features of the remembered stimulus (Fuster and Jervey, 1981, 1982; Miyashita and Chang, 1988; Miller et al., 1993; Nakamura and Kubota, 1995; Naya et al., 2001; Sigala and Logothetis, 2002).

This simultaneous activation of the areas that are interconnected with the prefrontal cortex during working memory has inspired views that the prefrontal cortex does not represent a memory trace for a particular item *per se*, but rather an abstract representation, allocation of cognitive resources, the focus of attention, or other top-down signals (Cowan, 1988; Miller and Cohen, 2001; Hazy et al., 2006; Postle, 2006; D'Esposito, 2007). In this framework, the contents of memory may be represented in PPC and IT, instead. Evidence against this idea comes from memory tasks that require maintenance in memory of an original item through sequential presentation of distracting stimuli, such as the delayed match to sample task. Both object and spatial versions of this task have been developed. In the context of the object delayed-match-to-sample task, persistent discharges of IT neurons are interrupted by non-matching, distractor stimuli presented after the sample (Miller et al., 1993). Conversely, responses in the ventral prefrontal cortex are able to represent the actively remembered sample's feature throughout the trial regardless of the distractor stimuli displayed (Miller et al., 1996). Equivalent findings have been obtained in the posterior parietal cortex for the spatial delayed-match-to-sample task (Katsuki and Constantinidis, 2012). Posterior parietal discharges represent the most recent stimulus location and are disrupted by distracting stimuli (Constantinidis and Steinmetz, 1996). Prefrontal neurons are able to represent the location of the original stimulus held in memory even after the appearance of distractors, in various tasks (di Pellegrino and Wise, 1993; Qi et al., 2010; Suzuki and Gottlieb, 2013).

Most recent studies have somewhat qualified these findings, for example demonstrating that differences between IT/PPC and prefrontal neurons in their ability to generate persistent activity that survives distractors are qualitative rather than quantitative (Woloszyn and Sheinberg, 2009; Qi et al., 2010), and that prefrontal neurons may respond better to distractors than actively remembered stimuli, in some tasks (Jacob and Nieder, 2014; Qi et al., 2015a). Nonetheless, in the context of the working memory tasks reviewed in the preceding paragraph, performance of the task is simply not possible based on the activation of the posterior parietal or inferior temporal cortex alone. The link of prefrontal activation with performance of working memory tasks that involve sequential presentation of distracting stimuli is confirmed by human imaging studies, as well: prefrontal activation is predictive of errors when activity representing an initial item is not maintained, whereas parietal cortex is indiscriminately activated by behaviorally relevant stimuli and distractors, alike (Sakai et al., 2002). Accumulating studies ascribing different roles in the activity of prefrontal and parietal cortex in working memory (Jacob and Nieder, 2014; Qi et al., 2015a), and functions such as attention and categorization (Swaminathan and Freedman, 2012; Crowe et al., 2013; Ibos et al., 2013), raise the alternative possibility that prefrontal and PPC/IT cortex are specialized for different aspects of working memory, as well as other cognitive functions (Katsuki and Constantinidis, 2012).

An instance of such differentiation may be the reported role of the posterior parietal cortex in determining the capacity of working memory (Todd and Marois, 2004, 2005). Activation of parietal cortex revealed by fMRI best predicts the number of simultaneous items maintained in working memory, relative to both earlier areas and the prefrontal cortex (Todd and Marois, 2004). The single-neuron basis of the phenomenon is not clear, however. Persistent discharges in the prefrontal and posterior parietal cortex reveal few differences between the two areas and no obvious neural correlate that is present only in the posterior parietal cortex and could determine capacity (Buschman et al., 2011).

The primacy of prefrontal cortex in working memory behavior is perhaps most vividly demonstrated in inactivation studies. Cooling experiments, which reversibly inactivate the underlying cortex by lowering its temperature, demonstrate much greater decreases in memory performance in the ODR task after prefrontal than posterior parietal cooling (Chafee and Goldman-Rakic, 2000), even when the areas inactivated have similar delay period activity (Chafee and Goldman-Rakic, 1998). The results of these studies parallel the effects of reversible inactivation of the frontal eye fields via muscimol injections, which similarly produce a significant impairment in memory-guided saccade performance (Sommer and Tehovnik, 1997; Dias and Segraves, 1999). In contrast, modest or no impairment was observed after muscimol inactivation of the posterior parietal cortex (Li et al., 1999; Chafee and Goldman-Rakic, 2000; Wilke et al., 2012), even though posterior parietal inactivation produces consistent deficits in tasks that require attention or selection between multiple stimuli (Wardak et al., 2002, 2004; Liu et al., 2010; Wilke et al., 2012). Small lesions to the dorsolateral prefrontal cortex also produce impairment in working memory performance for remembered stimuli in the contralateral space, an effect termed a “mnemonic scotoma” (Funahashi et al., 1993a; Funahashi, 2015). Equivalent results from localized lesions of the posterior parietal cortex are not available.

### Visual cortex

In recent years, human imaging studies have been successful in decoding information held in memory from the visual cortex, including the primary (Harrison and Tong, 2009; Albers et al., 2013; Xing et al., 2013) and extrastriate visual cortex (Ester et al., 2013; Sreenivasan et al., 2014b), suggesting that these areas maintain the contents of working memory (Tong and Pratte, 2012). This extraction of information has been possible with Multi-Variate Pattern Analysis (MVPA), examining the simultaneous pattern of activation of multiple voxels to different task conditions; the overall levels of activity in visual cortex may not rise above baseline during working memory (Offen et al., 2009). Imaging studies have gone as far as to determine that the size of the primary visual cortex alone is the best predictor of working memory ability (Bergmann et al., 2016). Importantly, MVPA could not decode information from the prefrontal cortex, or could not fully account for behavioral performance in the task (Harrison and Tong, 2009; Sreenivasan et al., 2014b).

This negative finding of information failing to be decoded from the prefrontal cortex during working memory, despite the known activation of prefrontal neuron in similar tasks, is telling about the interpretative limitations of these results. A tacit assumption when comparing the results of MVPA analysis across different cortical areas is that the structure of the voxel (typically in the order of 3 × 3 × 3 mm) is equivalent in the primary visual and prefrontal cortex. This is definitely not the case. Unlike the precise topography of visual space in the primary visual cortex, no retinotopic map (or other overarching organizational principle) has been revealed in the prefrontal cortex (Constantinidis and Procyk, 2004). Sampling the prefrontal cortex with chronic arrays of micro-electrodes spaced at 0.4 mm of each other reveal that the same cortical location is represented multiple times across the surface, and with no obvious map of space (Leavitt et al., 2013; Kiani et al., 2015). Simultaneously recorded neurons with movable electrodes spaced as close as to 0.2 mm of each other reveal only a slight bias toward similar spatial preference among neighboring prefrontal neurons (Constantinidis et al., 2001a). Precise stimulus location information is therefore represented in an extremely fine spatial scale, with the entire visual hemifield possibly represented in prefrontal modules no large than 0.5 × 0.5 mm in surface (Constantinidis et al., 2001a). Voxels averaging cortical volumes an order of magnitude larger are thus likely to obliterate stimulus information and will predictably fail to decode the information held in working memory, even if this is robustly represented in the activity of prefrontal neurons.

A recent fMRI study has in fact been successful in retrieving features of remembered stimuli, the orientation of a grating, from the prefrontal cortex during working memory (Ester et al., 2015). Such information may be represented more coarsely across the surface of the prefrontal cortex, making it possible to decode from fMRI activation patterns. In any case, these results argue directly against models of working memory that postulate solely a top-down control role for the prefrontal cortex, and place feature storage networks in the visual cortex (Ester et al., 2015).

MVPA methods still yield undeniable positive findings of fMRI imaging in the visual cortex and it is important to consider the neural basis of this activity that yields information about the contents of working memory. Early visual areas do not generate persistent activity. A recent study comparing activity in three cortical areas in the same animals, required to remember the direction of motion of a random-dot display, found virtually no persistent discharges in visual area MT, but robust activation in parietal area MST, in addition to prefrontal persistent activation (Mendoza-Halliday et al., 2014). This suggests an abrupt generation of feature-selective persistent activity in areas beyond the visual cortex. On the other hand, a small percentage of V1 neurons exhibit suppressed levels of discharges during working memory, below background levels (Super et al., 2001). It is unclear, however, whether V1 activity can be predictive of behavior in working memory task as this modulation was present for both correct and incorrect trials (Super et al., 2001). Changes in levels of activity in V1 during working memory are likely due to top-down projections from higher associative cortices, since V1 activation appears first in superficial layers (Roelfsema, 2015). A key aspect of this phenomenon is that background levels of activity in V1 are relatively “quiet,” thus making it possible to capture the subtle backwash from higher cortical areas, while the higher cortical areas themselves may be too noisy to detect these small signals. fMRI activation may additionally be detecting pre-synaptic activation of V1 neurons from higher cortical areas (Logothetis and Wandell, 2004), which makes V1 activity even less likely to be the ultimate storage of working memory contents and determinant of working memory performance.

## Conclusions and unresolved questions

The role of prefrontal persistent activity in working memory has been the focus of renewed attention in the past few years. This interest has been spurred by the realization that other brain areas are also active during working memory maintenance, that persistent activity may be shaped by the demands of the task rather than merely be representing information, and that dynamic patterns of activity can represent information in working memory. These results have inspired alternative models of working memory maintenance in the brain.

In this review, we make the case that persistent activity in the prefrontal cortex is both necessary and sufficient to account for information held in memory, across a variety of tasks and experimental conditions. Prefrontal persistent activity is also present in working memory tasks that do not rely on spatial stimuli and can encode attributes of stimuli (such as direction of motion and shape) or task variables and rules. Computational models based on persistent activity can account for levels of performance and patterns of errors depending on neuronal discharges to a greater extent than any alternative models.

Phenomena like repetition suppression are likely to be generated by synaptic rather than spiking mechanisms and they appear to correlate with behavior. However, they can only account for a limited set of behaviors and memory functions. Similarly, rhythmic or otherwise dynamic patterns of activity across the population of prefrontal neurons may convey information about stimulus properties. Such patterns of activation are not incompatible with persistent activity, either. It is upon future research to determine whether a causal relationship exists between such mechanisms and working memory performance.

The prefrontal cortex is not the only area that represents working memory information. Posterior parietal and inferior temporal areas have been long known to be active during working memory, though they appear insufficient to sustain information, for at least some tasks. It remains an open question on whether these areas are specialized for different aspects of working memory performance, or if their activity supports the maintenance of working memory in a distributed network that requires the prefrontal cortex. Information decoded from the primary visual cortex but not in the prefrontal cortex in fMRI studies cannot rule out a prefrontal involvement in working memory due to interpretational limitations that have to do with the topography of stimulus representation in these areas. It remains unclear whether neuronal activity in primary visual cortex plays any role in determining working memory behavior. Future work should aim to resolve these issues.

## Author contributions

MR and CC conceptually developed and wrote this review.

### Conflict of interest statement

The authors declare that the research was conducted in the absence of any commercial or financial relationships that could be construed as a potential conflict of interest.
